# *FBN3* gene involved in pathogenesis of a Chinese family with Bardet-Biedl syndrome

**DOI:** 10.18632/oncotarget.21415

**Published:** 2017-09-30

**Authors:** Yun Wang, Abir Garraoui, Liuzhi Zeng, Mingying Lai, Fen He, Huaizhou Wang, Chongyi Jiang, Yulan Chen, Lanlan Dai, Ning Fan, Huanming Yang, Jianguo Zhang, Xuyang Liu

**Affiliations:** ^1^ Shenzhen Key Laboratory of Ophthalmology, Shenzhen Eye Hospital, Shenzhen University, Shenzhen, China; ^2^ BGI-Shenzhen, Shenzhen, China; ^3^ Laboratory of Human Molecular Genetics, Faculty of Medicine, University of Sfax, Sfax, Tunisia; ^4^ The First People's Hospital of Chengdu, Chengdu, China; ^5^ Beijing Institute of Ophthalmology, Beijing Tongren Eye Center, Beijing Tongren Hospital, Beijing Ophthalmology & Visual Sciences Key Laboratory, Capital Medical University, Beijing, China; ^6^ James D. Watson Institute of Genome Sciences, Hangzhou, China; ^7^ Shenzhen Key Laboratory of Neurogenomics, BGI-Shenzhen, Shenzhen, China

**Keywords:** Bardet-Biedl syndrome, retinitis pigmentosa, *FBN3*, whole exome sequencing

## Abstract

**Purpose:**

This study was designed to evaluate the molecular genetics of a Chinese family with Bardet-Biedl syndrome (BBS).

**Methods:**

All the family members underwent medical history evaluation, ophthalmologic and physical examinations. Whole exome sequencing was performed on two affected individuals and their parents. All variants were verified in all family members by PCR amplification and Sanger sequencing.

**Results:**

Patients in this family were diagnosed as Bardet-Biedl syndrome, with an inheritance pattern of autosomal recessive. Compound heterozygous mutations of the *FBN3* gene (c.3616G>A and c.6037C>T) were identified by whole exome sequencing. Results from Sanger sequencing showed co-segregation of these compound heterozygous mutations in the *FBN3* gene with BBS disease in the family.

**Conclusion:**

Novel compound heterozygous mutations c.3616G>A and c.6037C>T of *FBN3* were identified in all affected individuals but not in the unaffected family members. This is the first time to the best of our knowledge, that the *FBN3* gene is involved in the pathogenesis of BBS. This study will expand our understanding about the gene spectrum related to this genetically heterogeneous disorder.

## INTRODUCTION

Bardet-Biedl syndrome (BBS, MIM 209900) was first described in 1920 by Georges Bardet and Arthur Biedl [1] as a rare autosomal recessive ciliopathy with the primary features of rod-cone dystrophy and other non-ocular manifestations including intellectual defects, obesity, polydactyly, hypogonadism, or renal malformations. Hepatic fibrosis, diabetes mellitus, reproductive and developmental delay, growth retardation, dental defects, speech delays, or cardiovascular problems were considered as the secondary features of this disorder. Some patients may not present with all these primary features, but the diagnosis of BBS could also be made when at least two secondary features are observed simultaneously [2, 3].

In view of its wide-ranging clinical and genetic features, molecular diagnosis of BBS seems to be particular complicated and difficult. A large number of variations were identified as the genetics causative factors of this disorder. To date, more than 19 disease-causing genes of BBS (including *BBS1, BBS2*……and *BBS19*) were identified [4–7]. Only 80% BBS patients can be explained by specific genetics causes [8]. There are at least 20 coding exons in each yet known BBS genes averagely. To sequence all these exons by Sanger sequencing and analyze the sequencing data needs a lot of costs and manpower, and has a risk that unknown genes related with BBS could not be detected [4, 7, 9]. Whole exome sequencing (WES) has provided an effective and precise method for molecular diagnosis of BBS [3].

The current study was designed to find the molecular basis of BBS in two patients using WES. Novel compound heterozygous mutations (c.3616G>A and c.6037C>T) of the fibrillin-3 (*FBN3*) gene were identified in two affected siblings. Briefly, the *FBN3* gene is located on chromosome 19p13.3–p13.2 in a region of 85 kb and is composed of 66 exons that encode a 2,809-amino acid protein. *FBN3* is most highly expressed in human fetal tissues and also expressed in the human adult brain, eye, lung, adrenal glands, stomach and ovaries [10, 11]. The association between *FBN3* and BBS need to be further studied.

## RESULTS

Affected individuals underwent a complete physical examination and detailed ophthalmic examination. All primary and secondary BBS features were investigated as described in Table 1 and both patients fulfilled the diagnostic criteria of BBS having at least four primary or three primary and two secondary BBS features.

**Table 1:** The disease phenotype of patients III1 and III2 compared with the typical disease phenotype of BBS

	*Typical disease phenotype of BBS*	*III1*	*III2*
Ocular features	Poor visual acuity	HM	—
	Nystagmus, abnormal pupil, iris defects, color blindness	Nystagmus, blepharophimosis	Nystagmus, blepharophimosis
	Concentric visual field contraction	?	?
	Retinitis pigmentosa	Y	Y
	Cataracts and vitreous opacities	Turbidity of lens postsac	N
Systemic features	Mental retardation	Y	Y
	Obesity	Height:152cm; weight: 70 kg; BMI: 30	Height:154cm; weight: 55 kg; BMI: 23
	Renal abnormalities	N	N
	Synpolydactyly	N	N
	Sexual dysgenesis	Development normal of external genitalia, age of menarche was 12 and amenorrhea at age 21	Development normal of external genitalia, age of menarche was 15 and amenorrhea at age 20
	Others	Language disorder, dental irregularities	Language disorder, dental irregularities, congenital cardiovascular abnormalities

HM: hand movement; —: incompatibility.

**Figure 1:**
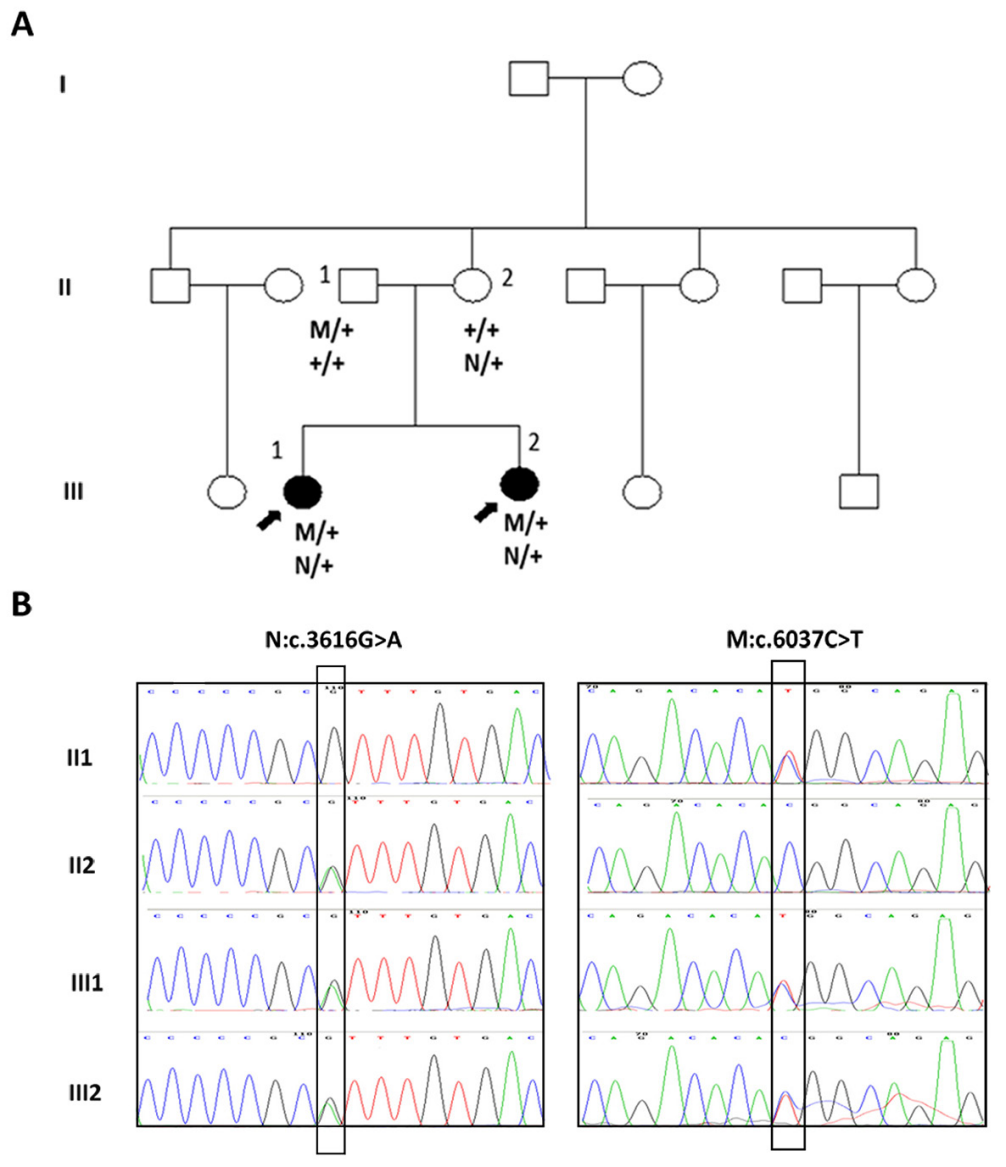
Mutations of the *FBN3* gene in a Chinese family with BBS syndrome **(A)** Pedigree of the family that had two daughters with BBS syndrome and the segregation of the two heterozygote mutations in family. **(B)** Sequence electropherograms of two affected individuals and their parents. Both patients III1 and III2 harbored compound heterozygous c.3616G>A and c.6037C>T of the *FBN3* gene. c.3616G>A of *FBN3* was carried by their mother II 2 while c.6037C>T mutation of *FBN3* was carried by the father II 1. In family pedigrees, roman numerals indicate generation number, arrows indicate probands, M and N stands for mutation identified, + is wild-type allele.

WES was applied to identify the pathogenic mutation of this family with BBS. 30379 SNPs and 1234 Indels were identified as potentially causative variants that shared by the two patients. Then, the sequence variants of the exome data were filtered against several public variation databases to reduce the number of potentially pathogenic mutations. First, those variants were checked to see if they are present in known BBS genes i.e BBS1-BBS19, and then the other genes were investigated. Filtering all exomes for a homozygous mutation causing the disease in the affected sibs (III1, III2), and which was present in heterozygous form in the unaffected mother and father (II1, II2). Variants satisfying a recessive homozygous inheritance model were not identified. This led us to investigate the possibility of recessive compound heterozygous inheritance. Under the hypothesis of a compound-heterozygous model, we filtered all exomes for variants present in the heterozygous state in all affected individuals for variants and also not present heterozygous in their mother’s exome. Then Sanger sequencing was used to confirm the exome data. The results showed that both patients were compound heterozygous for c.3616G>A (p.Val1206Ile) and c.6037C>T (p.Arg2013Trp) of the *FBN3* gene. The sequencing also revealed that the variation c.3616G>A was inherited from the mother while c.6037C>T from the father (Figure 1).

Polyphen-2 predicted p.Arg2013Trp of *FBN3* as “probably damaging” and SIFT predicted this mutation as “deleterious”. The valine residue at codon 1206 locates within an EGF-like core domain, a difference in the properties between the wild-type and mutant isoleucine amino acid residues, especially the lateral chain size, might disturb the core structure of this domain.

On the basis of the exome data analysis and Sanger sequencing, both patients III1and III2 harbored compound heterozygous c.3616G>A and c.6037C>T of the *FBN3* gene. The variant c.3616G>A was carried by their mother II 2 and c.6037C>T mutation was carried by the father II 1 (Figure 1B). The whole *FBN3* gene was sequenced in two siblings and their parents directly. Except for these two variants, ten polymorphisms of the *FBN3* gene including rs7246376, rs4804271, rs35840170, rs35579498, rs4804264, rs12975322, rs35306870, rs12150963, rs7245552 and rs7245429 were found in both patients III1 and III2. And the variant c.3616G>A of the *FBN3* gene were detected in one of the 101 normal controls enrolled in this study while the variant c.6037C>T were not found in any of the normal controls group. No other mutations in the *FBN3* gene were found in those two patients indicating that the compound heterozygous mutations c.3616G>A and c.6037C>T of the *FBN3* gene should be the cause of BBS in this family. To the best of our knowledge, this is the first time that *FBN3* was identified as the causative gene to BBS.

## DISCUSSION

In the current study, we report on two siblings with BBS, a rare, clinically and genetically heterogeneous disorder, in a non-consanguineous Chinese family. Both patients presented with mental retardation, speech and language disorder, dental irregularities, secondary amennorhea and ocular abnormalities including retinitis pigmentosa, blepharophimosis and nystagmus. To date, at least 19 BBS-related genes have been identified and mapped on different chromosomes. These disease-causing genes include *BBS1* (11q13), *BBS2* (16q21), *BBS3* / *ARL6* (3p12-q13), *BBS4* (15q22.3), *BBS5* (2q31), *BBS6*/*MKKS* (20p12), *BBS7* (4q27), *BBS8*/*TTC8* (14q32.11), *BBS9* / (*PTHB1*, 7p14), *BBS10* (12q21.2), *BBS11*/ *TRIM32*(9q33.1), *BBS12* (4q27), *BBS13 /MKS1* (17q23), *BBS14* / *CEP290* (12q21.3), *BBS15*/*C2ORF86* (2p15), *BBS16*/ *SDCCAG8*(1q43), *BBS17* / *LZTFL1* (3p21.31), *BBS18*/ *BBIP1/ BBIP10* (10q25.2) and *BBS19 / IFT27*(22q12.3). Most of the above genes are known to be involved in the formation and assembly process of a multiple subunit complex, called “BBSome” [12]. Some BBS subunits proteins including *BBS1*, *BBS2*, *BBS4*, *BBS5*, *BBS7*, *BBS8*, *BBS9* and *BBS18* play a role in the formation while some other subunits called the chaperonin-like proteins participate in the process of regulation, stabilization and assembly [12]. The BBSome is proposed to be responsible for transporting intracellular vesicles to the base of the cilia and therefore plays an important role in the ciliary function. Wei et al demonstrated that BBSome plays an essential role in the regulation of intraflagellar transport (IFT) assembly and turnaround in cilia [13]. IFT complex participates in the bidirectional motility of molecular assembly along the axoneme affecting formation, function and maintenance of cilia [14]. A defect in any part of the BBSome complex protein automatically may cause a mild or severe phenotype of BBS. However, the molecular mechanisms for this disease still remain unclear. Furthermore, all of the known BBS genes only can explain approximately 80% of the patients with BBS [8]. It is likely that other genes may be also involved in this disease.

In this study, WES was applied to identify genetic mutations in BBS patients, and revealed that the two patients were compound heterozygous for c.3616G>A (p.Val1206Ile) and c.6037C>T (p.Arg2013Trp) in the *FBN3* gene. Sanger sequencing confirmed the exome data and showed that c.3616G>A (p.Val1206Ile) and c.6037C>T (p.Arg2013Trp) of the *FBN3* gene carried by these siblings were inherited from their mother and father, respectively. Bioinformatic analysis by Polyphen-2 and SIFT proved these two substitutions pathogenicity. The compound heterozygous mutations of p.Val1206Trp and p.Arg2013Trp are novel mutations in the *FBN3* gene, which will be helpful in delineating the causative BBS mutations. Our study demonstrated, for the first time, the implication of the *FBN3* gene in BBS.

Fibrillin proteins are the most abundant components of extracellular microfibrils present in many connective tissues [15]. *FBN3* was discovered recently and has not been extensively studied [15]. It expressed in the cardiovascular system and the exocrine acini in pancreas. Fibrillin-3 is also found in the connective tissue of the central nervous system and the peripheral nervous system. In our study, both patients presented with symptoms include ocular abnormalities, mental retardation, hypoplasia (early menopause) and abnormal uterus by B ultrasound. These symptoms overlap with these two syndromes, Weill-Marchesani syndrome 1(WMS1) (MIM 277600) and polycystic ovary syndrome 1(PCOS1) (MIM184700), respectively. One of the major disease-causing genes of WMS1 is the fibrillin-1 (*FBN1*; MIM 134797), which was first identified with MFS in 1991 [16]. *FBN1* has overall homology of greater than 60% with *FBN3*, and contains multiple EGF-like domains. Linkage and immunohistochemical analyses strongly suggest a role for fibrillin-3 in the pathogenesis of PCOS [17–20]. All these indicated that the *FBN3* gene may also play an important role in pathogenesis of BBS.

In fact, *FBN3* deficiency is related to secondary amenorrhea because of its role in the pathogenesis of polycystic ovary syndrome. *FBN3* deficiency is also related to the congenital cardiovascular abnormalities in patient III1and hyperlipidemia and insulin resistant diabetes mellitus in patient III2 respectively. These findings may explain in part the clinical abnormalities noticed in patients in this family.

Up to date, there is no report showing that these two proteins, BBSome and Fibrillin-3, are structurally or functionally related. Further studies are needed definitely to detect the possible links between these two proteins and their role in the molecular basis for this disease.

In conclusion, we reported a Chinese BBS family and identified novel compound heterozygous mutations c.3616G>A and c.6037C>T in the *FBN3* gene in the two affected siblings. To the best of our knowledge, this is the first study indicating the association between the mutated *FBN3* gene and the pathogenesis of BBS. More families or cases should be studied to further our understanding of the *FBN3* gene associated molecular basis for BBS.

## MATERIALS AND METHODS

### Subjects

A non-consanguineous BBS family with two affected individuals (Figure 1A) was recruited in Shenzhen Eye Hospital. Written informed consent was obtained from the guardians of the patients according to the principles of Declaration of Helsinki. All 101 individuals in the control group were healthy and with no family history of BBS. This study was approved by Institute Review Board of Shenzhen Eye Hospital.

### Clinical examination

**Figure 2:**
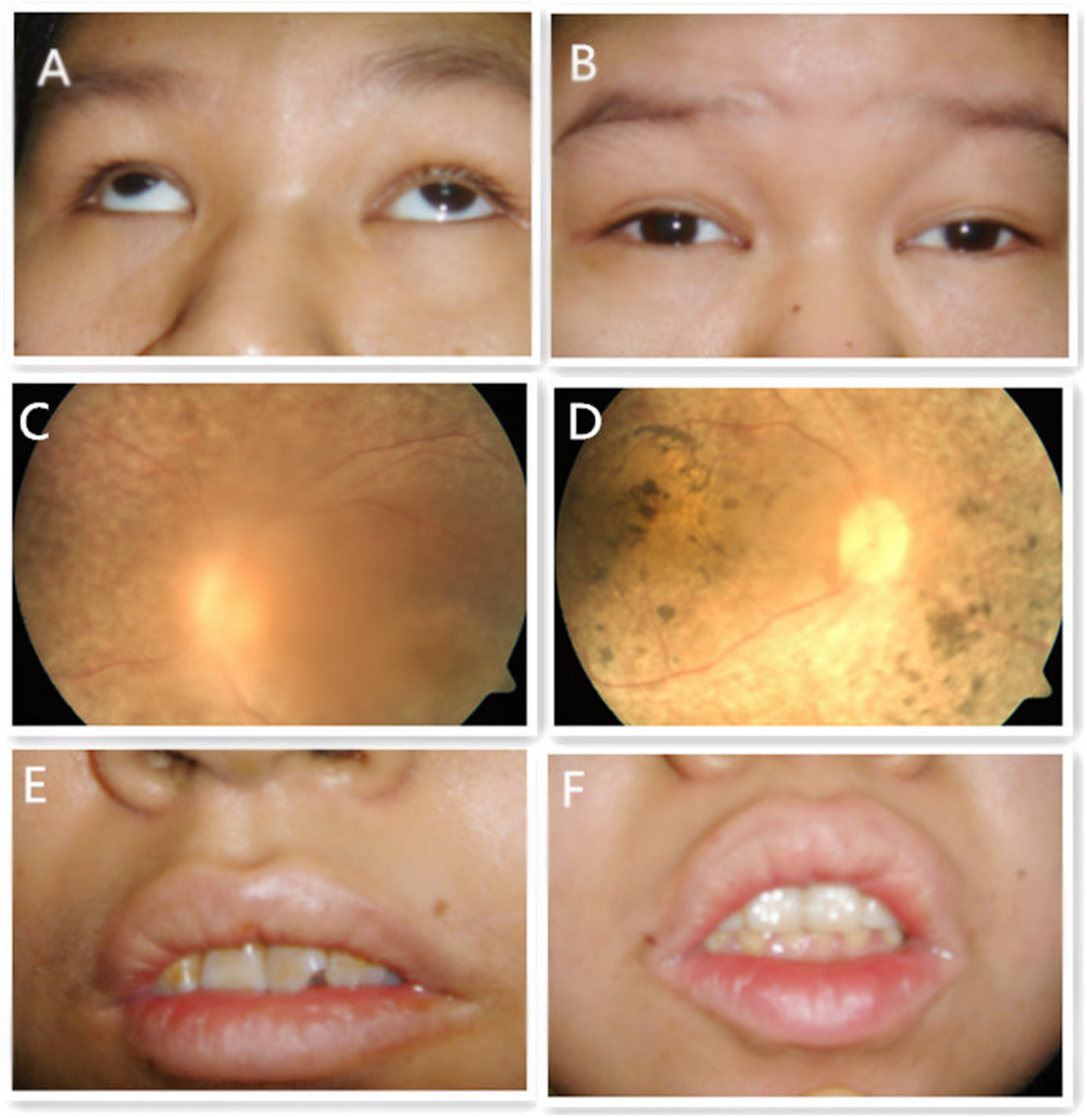
Eye appearance photography, fundus photography and dental appearance of patients III1 and III2 **(A)** (III1): Small eyelid fissure, nystagmus accompanied by strabismus; **(B)** (III2): Small eyelid fissure with nystagmus; **(C)** (III1) and **(D)** (III2): Retinal pigment degeneration and macular degeneration; **(E)** (III1) and **(F)** (III1): Irregular teeth arrangement.

Affected individuals underwent a complete physical examination and detailed ophthalmic examination. Patients III1 were 21 years old while her sister III2 was 23. Both patients presented with poor visual acuity including retinitis pigmentosa, macular degeneration, cataract and nystagmus (Figure 2). Both were obese (body weight of III1/III2 was 70 kg/52kg, the height of III1/III2 was 1.52/1.54 meters and the body mass index of III1/III2 was 30/23) and development with normal genital and normal of external genitalia with the age of menarche was 12(III1)/15(III2) and Amenorrhea at age 21(III1)/20(III2). Both showed with language disorder, mental retardation, dental irregularities and microsomia. III1 suffered from congenital cardiovascular abnormalities while III2 had total body complicated with hyperlipidemia and insulin resistant diabetes mellitus.

Auxiliary examination of Patients III1 and III2 showed that the level of plasma total cortisol (PTC) was decreased and glutamic-pyruvic transaminase (ALT) was elevated and the thyroid stimulating hormone 3 (TSH3) was increased. The uterus was smaller than normal with B mode Ultrasonography. Evoked potential report by electromyogram (EMG) showed that, the early component of differentiation in the left and right side was low by the limb somatosensory evoked potential (SEP) and the late component in the P40-N60 amplitude was increased. The cortical lesions cannot be excluded. It cannot be completed in the P300 amplitude and the target stimulus response time was significantly prolonged. It indicated that the perceptual disturbance, directional movement disorder and cognitive impairment cannot be excluded.

All primary and secondary BBS features were investigated (Table 1) and both patients fulfilled the diagnostic criteria of BBS having at least four primary or three primary and two secondary BBS features.

### DNA extraction

Blood samples from the two patients (III1, III2) and their parents (II1, II2) were collected in EDTA Vacutainers and processed. Genomic DNA of each individual was isolated and extracted from the peripheral blood (200μl) with the use of the QIAamp DNA blood mini kit (QIAGEN, Hilden, Germany) by standard protocols. DNA quantity and integrity was assessed by 1% agarose gel electrophoresis (AGE).

### Whole exome sequencing

#### Exome sequencing

Qualified genomic DNA extracted from the affected siblings and their parents was randomly sheared by sonication and then hybridized to SureSelect Human All Exon V1 for enrichment. The enriched exome was sequenced on the HiSeq 2000 to get paired end reads with each length of 90bp.

#### Alignment and variant calling

The sequencing reads were aligned to the human reference genome (GRCh37) with SOAPaligner and BWA separately. SOAPsnp v1.05 was then applied to the alignment resulted from SOAPaligner and GATK v3.3-0 was applied to alignment produced by BWA to call variants. Variants from these two pipelines were compared to each other in order to both increase our confidence in the called sites and to lower the false negative.

#### Analysis of the identified variants

Variant Effect Predictor (VEP, http://www.ensembl.org/Tools/VEP) tool was applied to determine the effect of the identified variants. We supposed that the splice acceptor and donor site mutations, non-synonymous variants, and short, frame-shift coding insertion and deletions were more likely to be the pathogenic mutation. Therefore, we focus on these kinds of mutations which were also satisfied co-segregation within the sequenced individuals.

### Verification of variants

Sanger sequencing was performed on all individuals to verify whether the potential candidate variants detected by WES were co-segregated with the disease phenotype in this family. Primer pairs flanking each candidate loci were designed by Primer Premier 5.0 according to the genomic sequences of Human Genome (UCSC hg19, NCBI build 37) and synthesized by BGI (BGI-Shenzhen, Guangdong, China). Direct polymerase chain reaction (PCR) was performed in a 30 μl reaction system. After evaluated by 1% agarose gel electrophoresis, the amplified products were then purified with a cycle-pure DNA kit (OMEGA; Bio-Tek, Doraville, GA). DNA sequencing was performed on an ABI-377XL automated DNA sequencer (Applied Bio systems, Foster City, CA). DNA sequences were assembled, aligned and analyzed by the DNAStar (Madison, WI, USA) software package with a genomic reference sequence. The description of the sequence variants were in accordance with the nomenclature guidelines recommended by the Human Genomic Variation Society (HGVS). To evaluate whether novel variants were either disease-associated mutations or benign polymorphisms, Sanger sequencing was performed on the corresponding region of the 101 control individuals. DNA sequencing was also carried out on the whole sequence of *FBN3* gene in two siblings and their parents to conform whether there are other variants may participate in the disease process. The potential effect of the missense mutations were predicted using SIFT (http://sift.jcvi.org/), and Polyphen-2 (http://genetics.bwh.harvard.edu/pph2/) tools.
